# Psychometric Properties of the Mindfulness Inventory for Sport (German Version)

**DOI:** 10.3389/fpsyg.2022.864208

**Published:** 2022-05-24

**Authors:** Alissa Wieczorek, Karl-Heinz Renner, Florian Schrank, Kirstin Seiler, Matthias Wagner

**Affiliations:** ^1^Department of Human Sciences, Institute for Sport Sciences, Universität der Bundeswehr München, Neubiberg, Germany; ^2^Department of Human Sciences, Institute for Psychology, Universität der Bundeswehr München, Neubiberg, Germany

**Keywords:** mindfulness, awareness, acceptance, focus, sports performance

## Abstract

Mindfulness-based training programs are highly established in competitive and recreational sports. One of the best-known approaches is the Mindfulness-Acceptance-Commitment Approach (MAC) by Gardner and Moore), which integrates mindfulness aspects of awareness, non-judgmental attitude, and focus. Based on these aspects, Thienot and colleagues developed and validated an English language sport-specific questionnaire, the so-called Mindfulness Inventory for Sport (MIS), for the assessment of mindfulness skills in athletes. The aim of this study is to psychometrically test a German language version of the MIS (MIS-D). To assess the psychometric properties, the MIS-D was examined in an online survey with an integrated test–retest design (*n* = 228) for reliability (internal consistency; test–retest reliability), validity (factorial; convergent), and measurement invariance (gender; competition type). The present results support the psychometric quality of the German language version of the MIS. Necessary replications should among others focus on checking the measurement invariance for further relevant subgroups.

## Introduction

Mindfulness has gained great interest in the scientific literature, which can be seen in an exponential growth trajectory of articles since the early 2000s (van Dam et al., 2018). Publications in the field of (sports) psychology cover all kinds of areas reaching from conceptualization, psychological theories to basic and applied science of mindfulness (Schindler, 2020).

Buddhist traditions are often seen as a substantial source and inspiration to mindfulness, especially with respect to the clinical context regarding most commonly evaluated therapeutic approaches like the mindfulness-based stress reduction (MBSR; Kabat-Zinn, 1990) or the mindfulness-based cognitive therapy (MBCT; Segal et al., 2002). Today’s understanding of mindfulness, which is inevitably linked to those clinical techniques, can be described “(…) as a kind of non-elaborative, non-judgmental, present-centered awareness in which each thought, feeling, or sensation that arises in the attentional field is acknowledged and accepted as it is.” (Bishop et al., 2004, 232).

Within this context, Bishop et al. (2004) proposed a two-component model of mindfulness to establish an operational definition. Those two components combine aspects of (1) self-regulation of attention and (2) orientation to experience. The first component includes the ability to sustain or switch the focus of attention and to inhibit secondary elaborative processing of thoughts, feelings, and sensations in the stream of consciousness. In addition, it is aimed to develop a so-called “beginner’s mind” in order to widen the own experience and get into a state of direct observation, which is not filtered through own beliefs, assumptions, expectation, or desires (Bishop et al., 2004). The second component refers to the aspect of dispositional openness, which describes a non-judgmental attitude of curiosity and receptivity to new experiences. In adopting this stance of curiosity and acceptance less behavioral/cognitive strategies and improved affect tolerance are expected, allowing any thoughts, emotions, and sensations to occur without further elaboration (Bishop et al., 2004; Thienot et al., 2014; Jansen et al., 2019).

In the context of sports, mindfulness is often characterized as a relevant component or psychological technique, respectively, in promoting high performance (Birrer et al., 2012). Due to the fact that mindfulness is assumed to be a multifaceted concept (van Dam et al., 2018), interventions can influence psychological functioning of athletes *via* numerous impact mechanisms like attention, self-regulation, or attitude (Birrer et al., 2012). Gardner and Moore (2004, 2007) developed one of the best-known mindfulness-based intervention programs in sport called MAC (Mindfulness-Acceptance-Commitment Approach). The authors hypothesize that efforts at internal self-control, task-irrelevant focus of attention, and restrictions in behavior that often accompany performance dysfunction can be replaced by a mindful stance of awareness, attention, and acceptance of internal processes. Therefore, this intervention aims to improve quality of practice, competitive performance, and enjoyment of athletic experiences. It is based on its three components (1) awareness of current thoughts, emotions, and bodily sensations, (2) acceptance/non-judgmental attitude toward current thoughts, emotions, and bodily sensation, and (3) commitment toward goal-relevant actions by maintaining goal-relevant actions for a greater behavioral flexibility. Numerous mindfulness-based interventions were added in the past years, such as MSPE (Mindful Sports Enhancement Program) by Kaufman et al. (2009), MMTS (Meditation Training for Sport) with a focus on meditation (Baltzell and Akthar, 2014), MTC (Mindfulness Training for Coaches) with coaches as target group (Longshore and Sachs, 2015), or MAIC (Mindfulness-Acceptance-Insight-Commitment) especially for Chinese athletes (Si et al., 2016)—just to name a few.

All mentioned approaches compromise the substantial elements of mindfulness, namely, present-focused awareness and attention, non-judgmental and accepting approach to situations, physical sensations, and emotions, as well as openness and curiosity toward experience, and last compassion for self and others (Zizzi, 2017).

As a consequence of this increasing prevalence of mindfulness-based interventions in sport, a context-specific instrument is needed to assess mindfulness skills among athletes and especially, the effectiveness of the numerous interventions. In order to meet these demands, Thienot et al. (2014) developed an English questionnaire that enables to underpin those mindfulness-based programs aiming to enhance sport performance. The questionnaire is based on the above-mentioned model by Gardner and Moore (2007) and is therefore developed on the following three components: (1) awareness, (2) non-judgmental attitude, and (3) refocusing. While awareness is defined as “the ability to closely observe one’s internal experience like cognitions, emotions or bodily sensations in the present moment,” the non-judgmental attitude represents “the willingness to allow and accept one’s internal experience as it occurs without any attempt to judge and criticize oneself for experiencing these cognitions, emotions or bodily sensations.” As last component, refocusing is defined as “the ability to disengage from elaborative processing like distraction, rumination or worry in order to remain focused or to quickly refocus on task-relevant cues” (Thienot et al., 2014, 73–74).

The study design implemented by Thienot et al. (2014) to develop a respective context-specific instrument followed a three-stage approach (Netemeyer et al., 2003) to test reliability and validity. The validity aspect is grounded in Messick’s (1995) classification of construct validity into content, structural, generalizability, and external aspects. Therefore, in stage 1 an initial pool of domain-specific items capturing all three components was generated and rated by six external judges in order to assess the content aspect of validity. Based on the results of the expert review, ten items for each subscale were retained (Thienot et al., 2014). In stage 2, the structural aspects of construct validity (Messick, 1995) of the 30-item measure were examined and exploratory factor analysis revealed 19 items (eight awareness, six non-judgmental, five refocusing) loading on three distinct factors. Additional reliability analyses for these remaining items in each subscale showed acceptable evidence of internal consistency (Thienot et al., 2014). In stage 3, the focus of analysis was particularly on structural, generalizability, and external aspects of construct validity (Messick, 1995). A confirmatory factor analysis was conducted to refine the structure of the instrument and to remove problematic items. A 15-item (five awareness, five non-judgmental, five refocusing), three-factor measurement model, was identified that adequately fit the data. The generalizability aspect was assessed through measurement invariance testing for gender (male vs. female) and sport types (team vs. individual sports). Results provide only partial invariance between males and females, whereas evidence of measurement consistency across sport types could be detected for metric and scalar invariance. Finally, substantial correlations between the MIS subscales and five other measures (mindful trait in daily life, flow disposition, worry and concentration disruption, perfectionism, and rumination) were found in terms of evidence for the external aspect of construct validity (Thienot et al., 2014).

Mindfulness as a construct takes over different roles in the sports context and can therefore act as a predictor as well as a mediator or moderator between performance predictors and performance itself and in terms of this knowledge as an outcome of respective interventions (Noetel et al., 2019a). Consequently, reliable and validate assessment tools, such as the MIS, are assumed to be beneficial for a variety of different research questions in the context of mindfulness in sports. The promising work provided by Thienot et al. (2014) underpinned the request of a German version of the respective original questionnaire. Therefore, the aim of this paper is to examine the psychometric properties of the MIS-D (German translation). Regarding reliability, it is assumed that the MIS-D items reflecting the same construct will yield a strong coherence (internal consistency, hypothesis 1). Moreover, it is hypothesized that the MIS-D will provide similar results when repeated in the same sample within a six-week interval (test–retest reliability; hypothesis 2). Regarding validity, it is assumed that the postulated three-factor structure (1. awareness/2. non-judgmental/3. refocusing) can be identified (factorial validity, hypothesis 3) and that these factors will prove to be equivalent across gender and competition type (measurement invariance, hypothesis 4a/b). In terms of convergent validity (hypothesis 5), substantial correlations between the three MIS subscales and five conceptually related variables like mindfulness in daily life, flow, worry and concentration disruption, perfectionism, and rumination are expected.

## Materials and Methods

### Participants

A total of 228 female and male adults (63 females, 165 males) with a mean age of *M* = 23.42 years (*SD* = ± 2,91 years; range: 16–33 years) participated in this study. Participants were recruited online within the author’s institutional context during February and August 2021. The total sample was used for the evaluation of internal consistency (hypothesis 1), factorial validity (hypothesis 3), and measurement invariance across gender and competition type (hypothesis 4a/b). For the test–rest reliability (hypothesis 2) a subsample of 106 adults (28 females, 78 men) with a mean age of 23.31 years (*SD* = ± 2.84 years; range: 18–31 years) was available. For the analysis of convergent validity (hypothesis 5) the three subscales of the MIS-D (awareness, non-judgmental attitude, refocusing) were correlated with five conceptually related variables (mindful trait in daily life; flow; worry/concentration disruption; perfectionism; rumination) based on the different subsamples as not all subjects completed all questionnaires in the second survey phase after 6 weeks (*Mindful trait in daily life:* 211 participants, 59 females, 152 males; *M_age_* = 24.46 years, *SD_age_* = ± 2.99 years, range: 16–33 years; *Flow disposition:* 228 participants, 63 females, 165 males, *M_age_* = 23.42 years, *SD_age_* = ± 2.91 years, range: 16–33 years; *Worry and concentration disruption:* 92 participants, 27 females, 65 males; *M_age_* = 23.12 years, *SD_age_* = ± 2.88 years, range: 13–32 years; *Perfectionism*: 208 participants, 59 females, 149 males, *M_age_* = 24.49 years, *SD_age_* = ± 3.00 years, range: 16–33 years; *Rumination*: 221 participants, 62 females, 159 males, *M_age_* = 23.43 years, *SD_age_* = ± 2.94 years, range: 16–33 years).

### Measures

#### Mindfulness in Sport

The 19-item MIS-D (German translation), which results from the translation of the English original version, was developed out of an independent back translation and includes eight awareness items, six non-judgmental items, and five refocusing items (see Supplementary Material). A 6-point Likert scale (1 = *not at all*; 6 = *very much*^1^) was used to indicate how much each statement reflects the participants’ experience following the instruction: “The statements below describe a number of things that athletes may experience just before or during their sport performance. Please circle the number that best indicates how much each statement is generally reflective of your recent experience.^2^” (Thienot et al., 2014, 75) The respective format was chosen following the Mindfulness Attention and Awareness Scale (MAAS), representing the most common instrument in literature assessing mindfulness in non-athletic settings (Brown and Ryan, 2003; Grossman, 2008).

The German instruments to test for convergent validity (Campbell and Fiske, 1959) were selected in accordance with the original article by Thienot et al. (2014) and are presented in the following section as well as further refined hypotheses (hypotheses 5a–5f).

#### Mindful Trait in Daily Life

The German version of the Mindfulness Attention and Awareness Scale (MAAS) was used in order to assess the tendency to be mindful in daily life (Michalak et al., 2008). Therefore, the disposition to what extent internal and external conditions of the present moment are fully perceived are primarily assessed. For this purpose, 15 items formulated in a “mindless” way are rated on a 6-point Likert scale (1 = *almost always*; 6 = *almost never*^3^). According to Brown and Ryan (2003) states of low mindfulness are more perceptible and less likely to produce falsely positive responses in the sense of being attentive and therefore better suited for the assessment of mindfulness than positively formulated items. The internal consistency of the German version (*α* = 0.83) is almost identical to the English scale (*α* = 0.82). Like Thienot et al. (2014) already postulated, substantial positive correlations in terms of convergent validity between the MAAS and scores of the MIS awareness and refocusing scale are expected (hypothesis 5a).

#### Flow Disposition

The 10-item Flow Short Scale (FSS; Flow-Kurzskala) by Rheinberg et al. (2003) was used in order to assess the tendency to experience flow states while performing in sport. This questionnaire measures the components of a flow experience as described by Csikszentmihalyi (1975) on a 7-point Likert scale (1 = *not at all*; 7 = *very much*^4^). In general, the concept of flow represents a temporary psychological state of optimal experience, that emerges when the athlete’s skill level in balance with the respective challenge (Csikszentmihalyi, 2009). The FSS is no context-specific instrument and can be applied to obtain a typical flow score for different kinds of actions or situations, such as sports performance. Three optionally items covering worry and anxiety were excluded as these items are covered by a separate questionnaire of the following reference construct *worry and concentration disruption*. In addition, the Short Dispositional Flow Scale (Short DFS-2; (Jackson et al., 2008) used by Thienot et al. (2014) includes no such items. The 10-item FSS shows high evidence of internal consistency with Cronbach’s alpha around 0.90 (Rheinberg et al., 2003). As already mentioned in the original paper, mindfulness and flow share conceptual similarities as both focus on present moment focus of attention (Aherne et al., 2011; Moran, 2012). The effect of mindfulness-based approaches for performance enhancement in the sports and exercise domain is directly linked to the flow phenomenon as demonstrated by Sappington and Longshore (2015) or Noetel et al. (2019b). Additionally, Jekauc and Kittler (2015) propose an effect mechanism of mindfulness in sports, that leads to higher performance through (1) development of a flow state, (2) improved maintenance and regulation of concentration/attention, or (3) emotion regulation. Consequently, a substantial positive correlation in terms of convergent validity between all three subscales of the MIS-D and the obtained flow score with the FSS is expected (hypothesis 5b).

#### Worry and Concentration Disruption

The tendency to worry and be distracted just before and during competition was originally assessed by the Worry and Concentration Disruption subscales of the Sport Anxiety Scale 2 (SAS-2, (Smith et al., 2006). A German equivalent to this questionnaire is the so-called Wettkampfangstinventar-Trait (WAI-T) by Brand et al. (2009) which captures the following three components: (1) somatic anxiety, (2) worry, and (3) concentration disruption. Each component has four items and is rated on a 4-point Likert scale (1 = *not at all*; 4 = *very much so*^5^). Using an athletes sample, Brand et al. (2009) reported internal consistencies reaching from *α* = 0.811 (somatic anxiety), *α* = 0.826 (worry) to *α* = 0.768 (concentration disruption). As part of the operational definition by Bishop et al. (2004), the component *self-regulation of attention* emphasizes that mindfulness practices aim to improve cognitive inhibition, especially at the level of stimulus selection. This means that secondary elaborative processing of thoughts, feelings, and sensations that arise in the stream of consciousness should be inhibited. The positive promotion effect of mindfulness-based interventions on performance-related psychological parameters like sport anxiety-related worry or task-related worries could be shown in several studies (de Petrillo et al., 2009; Thompson et al., 2011). As a consequence, a substantial negative correlation in terms of convergent validity between the MIS-D subscales and the worry and concentration disruption subscale of the WAI-T is expected (hypothesis 5c).

#### Perfectionism

According to Hewitt and Flett (1991), central determinants of perfectionism are setting unrealistically high standards, selective attention to mistakes and their overgeneralization, constant evaluation of one’s own actions, and the tendency to “all-or-nothing thinking” in the sense that only absolute success or total failure is possible. Within the original paper a brief version of the Hewitt and Flett (1991) Multidimensional Perfectionism Scale (HF-MPS) and the Frost et al. (1990) Multidimensional Perfectionism Scale (F-MPS) by Cox et al. (2002) was used to measure perfectionism. In reference to that, the German version of the F-MPS by Altstötter-Gleich and Bergemann (2006) with six subscales and 35 items rated on a 6-point Likert scale (1 = *strongly disagree*; 6 = *strongly agree*^6^) was used. In a sample of 1,170 participants, the internal consistency of the subscales ranged from *α* = 0.70 (Doubts about Action) to *α* = 0.90 (Organization). For several multidimensional instruments assessing perfectionism, second-order factors like *positive striving* vs. *maladaptive evaluation concerns* (Frost et al., 1993; Slaney et al., 1995), *adaptive* vs. *maladaptive perfectionism* (Rice et al., 1998) or *healthy* vs. *unhealthy perfectionism* (Stumpf and Parker, 2000) were identified and labeled as *functional* vs. *dysfunctional components* (Altstötter-Gleich and Bergemann, 2006). Based on these findings, a substantial negative correlation in terms of convergent validity between all MIS-D subscales and the dysfunctional components (Concern Over Mistakes, Doubts About Action, Parental Expectations, Parental Criticism) is expected (hypothesis 5d) due to the fact that higher mindfulness skills are associated with lower maladaptive perfectionism (Hinterman et al., 2012). Further, Hinterman et al. (2012) found a negative correlation between functional/positive perfectionism and the subscale *Accept without judgment* of the Kentucky inventory of mindfulness skills (KIMS; Baer et al., 2004). Consequently, a substantial negative correlation in terms of convergent validity between the non-judgmental subscale of the MIS-D and the functional/positive perfectionism items of the German F-MPS (Personal Standards, Organization) is expected (hypothesis 5e).

#### Rumination

Rumination pertains to self-attentiveness motivated by perceived threats, losses, or injustices to the self (Trapnell and Campbell, 1999). The tendency to ruminate was assessed by the German Rumination Scale (König, 2012) of the original Rumination Reflection Questionnaire (RRQ) by Trapnell and Campbell (1999). This questionnaire uses a 5-point Likert scale (1 = *strongly disagree*; 5 = *strongly agree*^7^). Contrarily, Thienot et al. (2014) used the Rumination subscale of the Emotion Control Questionnaire-2 (Roger and Najarian, 1989) which was developed with a True/False response format. Psychometric analyses of the German Rumination Scale of the RRQ showed an internal consistency of *α* = 0.904 based on sample of 577 subjects with different mental impairments (D. König-Teshnizi, personal communication, January 20, 2021). Like Hinterman et al. (2012) could already establish a negative correlation between mindfulness and perfectionism, a similar finding emerged for the correlation between mindfulness and rumination (Josefsson et al., 2017). Therefore, a substantial negative correlation in terms of convergent validity was particularly expected between the German Rumination Scale and the MIS-D refocusing and non-judgmental scale in our study (hypothesis 5f).

### Procedure

Participants completed the 19-item German version of the Mindfulness Inventory for Sport (MIS-D), that was developed in stage 2 of the respective validation process by Thienot et al. (2014). The protocol was approved by the institutional review board prior to data collection. The questionnaire package was completed online *via* Social Science Survey (SoSci) consisting of an upstream information about the nature of the study, the right to withdraw, the storage and confidentiality of collected data, and a digital declaration of consent as obligatory requirement for participation, followed by selected questions on personal and sporting data in accordance to the Motorik-Modul Physical Activity Questionnaire (MoMo-PAQ; Woll et al., 2011), the MIS-D and the five conceptually related variables.

### Statistical Analysis

Subsequent results on reliability, validity, and measurement invariance of the MIS-D (German translation) were calculated using SPSS 27.0 (IBM Corp., Released 2020) and R 4.1.1 (R Core Team, 2021). The significance level for all statistical tests (two-tailed) was set *a priori* to *α* = 0.05.

For the evaluation of internal consistency of all three MIS-D subscales (hypothesis 1), Cronbach’s Alpha as well as McDonald’s Omega were calculated (Briggs and Cheek, 1986; Streiner, 2003; Berger, 2019). Intraclass correlation coefficient (ICC) was used to evaluate test–retest reliability (hypothesis 2) using a mean of multiple measurement, absolute agreement 2-way mixed-effects model (Koo and Li, 2016).

To examine factorial validity of the three MIS-D subscales (hypothesis 3), a confirmatory factor analysis (CFA, reflective model) was conducted. Considering the given discrete response categories of the MIS-D, the WLSMV discrepancy function (Weighted Least Squares Mean and Variance adjusted) was chosen as an appropriate and robust estimation method (DiStefano and Morgan, 2014; Li, 2016). To evaluate global model fit, fit indices including Root Mean Square Error of Approximation (RMSEA; ≤0.06), Standardized Root Mean Residual (SRMR; ≤0.08), Comparative Fit Index (CFI; ≥0.90), and Tucker–Lewis Index (TLI; ≥0.90) were calculated, based on recommendations by Hu and Bentler (1999) and Schermelleh-Engel et al. (2003). RMSEA is attributed with a salient meaning in confirmatory context and therefore plays a superordinate role in assessing global model fit (Bentler and Bonett, 1980; Hu and Bentler, 1999; Schermelleh-Engel et al., 2003; Bühner, 2011). As structural equation models and their substructures can be problematic despite a good global fit, the model’s local fit was assessed additionally. Therefore, inner-method convergent and divergent measures like indicator reliability (IR), factor reliability (FR), average variance extracted (AVE), Fornell–Larcker ratios (FLR), and a scaled *χ*^2^ difference test statistic (Satorra, 2000; Satorra and Bentler, 2001) for robust estimation methods were applied, based on recommendations by Kline (2016).

Measurement invariance across gender and competition type of the MIS-D (hypothesis 4a/b) were examined by testing and comparing nested models using multiple-group analysis with each successive model including the previous model restrictions. As nested models are not independent of each other, the decision whether to accept or reject a model was initially based on the *χ*^2^ difference test (Hu and Bentler, 1999), respectively the Satorra–Bentler scaled *χ*^2^ (Satorra, 2000; Satorra and Bentler, 2001). However, since the *χ*^2^ value depends on the sample size, Cheung and Rensvold (2002) and Meade et al. (2008) suggest using the CFI difference test instead. According to this test, if a difference in CFI of 0.02 between the base model and the respective model is exceeded, no significant difference between those two models can be detected. Common fit statistics like RMSEA (Root Mean Square Error of Approximation; ≤ 0.06), CFI (Comparative Fit Index; ≥ 0.90), and TLI (Tucker–Lewis Index; ≥ 0.90) were used for further indications of model validity (Hu and Bentler, 1999; Schermelleh-Engel et al., 2003).

For the evaluation of convergent validity (hypothesis 5), we calculated Bravais–Pearson correlation coefficients between the MIS-D subscales awareness, non-judgmental attitude, and refocusing and the five conceptually related variables (one’s tendency to be mindful in daily life, flow, worry/concentration disruption, perfectionism, and rumination).

## Results

### Reliability

All three MIS-D subscales (*N* = 228) showed adequate internal consistency according to Cronbach’s alpha (hypothesis 1; awareness: 0.725; non-judgmental: 0.721; refocusing: 0.738). This goes along with an optimal level of homogeneity represented by mean interitem correlations ranging from 0.250 to 0.371. To address the fact that items do not always capture a construct homogeneously, McDonald’s Omega was calculated as a complement to Cronbach’s Alpha (awareness: 0.729; non-judgmental: 0.726; refocusing: 0.745), even though both measures show only marginal estimate differences. The MIS-D (German translation) provides moderate to good test–retest reliability (hypothesis 2; interval: 6 weeks; *n* = 106; *ICC_awareness_* = 0.58, 95% CI [0.39, 0.72], *p* = 0.000; *ICC_nonjudgmental_* = 0.77, 95% CI [0.66, 0.84], *p* = 0.000; *ICC_refocusing_* = 0.79, 95% CI [0.70, 0.86], *p* = 0.000). In summary, results provide good evidence for the reliability of the MIS-D (German translation).

### Factorial Validity

**Figure 1:**
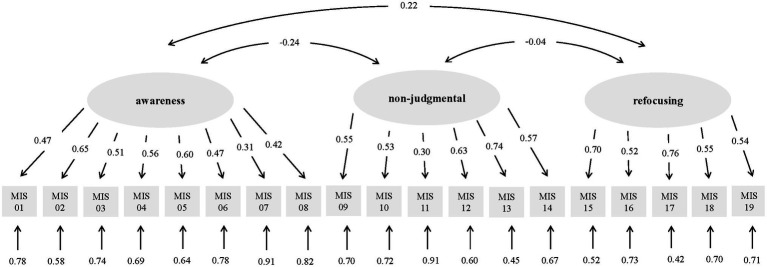
Factorial validity of the MIS-D (German translation). Results of the confirmatory factor analysis (standardized solution).

To assess factorial validity (hypothesis 3), WLSMV estimation was applied with regard to discrete response categories. The proposed three-factor structure encompassing eight indicators for awareness, six indicators for refocusing and five indicators for refocusing (see Figure 1) reached good global model fit with *χ*^2^ (149) = 201.520, *p* = 0.003, RMSEA = 0.039 with 90% CI [0.024, 0.053] and SRMR = 0.069. While the *χ*^2^/*df* ratio of 1.35 supports this assumption, CFI and TLI values of 0.898, respectively, 0.883 show marginal deviations from an adequate fit (≥0.90).

For inner-method convergent measures (see Table 1), each estimated factor loading differed significantly from zero (*p* < 0.001) and the factor reliability (FR) for all three latent variables clearly exceeded the required minimum of 0.60. By contrast, none of the latent variables could explain more than 50% of the variance (AVE) of its subordinated indicators and therefore no sufficient explanatory power could be identified. In addition, in only four out of 19 items, the variance that was assessed by the respective superordinate latent variable (IR) was higher than 40% and therefore a few indicators were proven to be particularly critical at this point.

**Table 1:** Inner-method convergent and divergent measures within MIS-D (German translation).

Subscales	Item No.	IR	*t*-value loading	FR	AVE	FLR	Scaled *χ*^2^ difference test statistics
Awareness	1	0.216	–	0.99	0.28	0.21	27.651***
	2	0.424	6.046***				
	3	0.264	5.750***				
	4	0.313	5.161***				
	5	0.357	7.499***				
	6	0.222	5.125***				
	7	0.094	3.117***				
	8	0.179	4.214***				
Non-judgmental	9	0.301	–	0.96	0.32	0.18	
	10	0.278	5.695***				
	11	0.091	3.415***				
	12	0.395	6.060***				
	13	0.553	7.143***				
	14	0.328	6.290***				
Refocusing	15	0.484	–	0.93	0.37	0.13	
	16	0.268	5.962***				
	17	0.582	8.995***				
	18	0.302	6.436***				
	19	0.289	6.870***				

***= *p* < 0.001; IR, indicator reliability; FR, factor reliability; AVE, average variance extracted; FLR, Fornell–Larcker ratio.

For inner-method divergent measures (see Table 1), the AVE of all three latent variables was higher than the maximum squared intercorrelation (MSI) resulting in FLRs lower than 1, which can be interpreted as an indication for good inner-method discriminance. Furthermore, the scaled difference *χ*^2^ test statistic proved that the proposed model can be retained (*χ*^2^*_scaled_* = 27.651***).

### Measurement Invariance Across Gender and Competition Type

Analyses of measurement invariance across gender and sports type of the MIS-D were conducted with the 19-item three-factor model. To test for measurement invariance of the MIS-D (hypothesis 4a/b), a multi-group comparison within the framework of confirmatory factor analysis for ordinal data was applied. The restricted models were compared with the basic model after gradually equating more and more parameters in an iterative manner (configural/metric/scalar/strict invariance) across gender (female, *n* = 63 vs. male, *n* = 165) and competition type (competition, *n* = 92 vs. non-competition athletes, *n* = 136).

Results on measurement invariance across gender of the MIS-D (hypothesis 4a; see Table 2) support the assumption of configural, metric, scalar, and strict invariance across gender, which means that the postulated three-factor structure displays equivalent in both groups and that males and females show a comparable item responsiveness (∆CFI < 0.02).

**Table 2:** Results of multi-group comparing within MIS-D (German translation).

	*χ* ^2^	df	*p*	**∆** *χ* ^2^	**∆**df	p(**∆***χ*^2^)	CFI	∆CFI	TLI	RMSEA
*Gender*										
Baseline males	190.808	149	0.012	-	-	-	0.876	–	0.858	0.041
Baseline females	151.575	149	0.426	-	-	-	0.977	–	0.973	0.017
Configural invariance	339.131	298	0.051	-	-	-	0.903	–	0.889	0.035
Metric invariance	350.692	314	0.075	15.199	16	0.510	0.914	|0.011|	0.906	0.032
Scalar invariance	369.959	330	0.064	33.082	32	0.414	0.906	|0.003|	0.903	0.033
Strict invariance	388.369	349	0.072	52.342	51	0.422	0.908	|0.005|	0.909	0.032
*Competition type*										
Baseline competition	183.887	149	0.027	–	–	–	0.860	–	0.839	0.051
Baseline non-competition	177.547	149	0.055	–	–	–	0.877	–	0.859	0.038
Configural invariance	361.104	298	0.007	–	–	–	0.869	–	0.850	0.043
Metric invariance	378.321	314	0.007	18.259	16	0.309	0.866	0.003	0.855	0.043
Scalar invariance	420.564	330	0.001	52.818	32	0.012	0.812	0.057	0.805	0.049
Scalar invariance										
MIS19 ~ 1	414.664	329	0.001	48.397	31	0.024	0.822	0.047	0.815	0.048
Scalar invariance										
MIS19 ~ 1 + MIS14 ~ 1	409.574	328	0.001	44.439	30	0.043	0.831	0.038	0.823	0.047
Scalar invariance										
MS19 ~ 1 + MIS14 ~ 1 + MIS11 ~ 1	405.490	327	0.002	41.067	29	0.068	0.837	0.032	0.830	0.046

**χ*^2^ = chi square; df = degrees of freedom; *p* = level of significance of χ^2^test; ∆df = changes in degrees of freedom; p(∆*χ*^2^) = level of significance of scaled *χ*^2^test; CFI = Comparative Fit Index; ∆CFI = CFI difference test; TLI = Tucker–Lewis Index; RMSEA = Root Mean Square Error of Approximation*.

Results on measurement invariance across competition type of the MIS-D (hypothesis 4b; see Table 2) could not provide strong evidence of complete measurement invariance for the three-factor model across competition and non-competition athletes. Indices for the configural and metric model reflect an adequate fit (RMSEA ≤0.05) as well as ∆CFI less than 0.02 (metric model). As this pattern of ∆CFI (<0.02) could not be transferred to scalar invariance level, it is tested for partial invariance by freeing one item at a time (van de Schoot et al., 2012). In this case, a maximum of three items can be freed according to Dimitrov’s (2010) guidelines stating that less than 20% free parameters are acceptable. The following model modification was carried out on the basis of the largest improvement in the CFI by freeing the intercepts of item 19 (“When I become aware that some of my muscles are sore, I quickly refocus on what I have to do”), item 14 (“When I become aware that I am angry at myself for making a mistake, I criticize myself for having this reaction”) and item 11 (“When I become aware that I am really excited because I am winning, I think that is bad to have this feeling of excitement”). Although the intercepts of those three items were freed, no partial measurement invariance at scalar level could be shown as the CFI difference remains larger than 0.02. Therefore, findings of measurement invariance across the two competition type groups only support the assumption of configural and metric invariance.

### Convergent Validity

The following correlations of the MIS-D (German translation) subscales of awareness, non-judgmental attitude, and refocusing with the above-mentioned reference constructs emerged (see Table 3).

**Table 3:** Bivariate correlations (Bravais–Pearson correlation) between the MIS-D (German translation) and the subscales of five conceptually related constructs.

Subscales	*n*	*M*	*SD*	Skewness	Kurtosis	**α**	MIS-D	MIS-D Non-judgmental	MIS-D refocusing
MAAS	211	60.06	11.12	−0.34	−0.26	0.83	0.108	0.207^**^	0.213^**^
FSS	228	5.37	0.85	−0.36	−0.17	0.90	0.110	0.095	0.458^**^
WAI-T somatic anxiety	92	10.53	2.87	−0.02	−0.68	0.81	0.306^**^	−0.099	−0.147
WAI-T worry		9.62	2.80	0.13	−0.22	0.83	0.111	−0.265^*^	−0.059
WAI-T concentration disruption		6.12	1.93	1.16	2.09	0.77	0.076	−0.139	−0.250^*^
F-MPS functional	208	59.29	10.44	−0.71	0.29	–	0.126	−0.180^**^	0.142^*^
F-MPS dysfunctional		57.80	19.18	0.50	−0.43	–	0.076	−0.229^**^	−0.132
RRQ	221	41.23	9.44	−0.32	−0.72	0.90	0.088	−0.236^**^	−0.228^**^

^*^ = *p* < 0.05, two-tailed. ^**^ = *p* < 0.01, two-tailed. MAAS = Mindful Attention and Awareness Scale; FSS = Flow Short Scale; WAI-T = Wettkampfangstinventar-Trait; F-MPS = Frost et al. Multidimensional Perfectionism Scale; RRQ = Rumination Reflection Questionnaire; MIS-D = Mindfulness Inventory for Sport (German version).

#### Mindful Trait in Daily Life

The theoretically expected correlation between the MIS-D refocusing subscale and the MAAS (*r* = 0.213, *p* = 0.002, hypothesis 5a) was similar to Thienot et al. (2014). In accordance with Thienot et al. (2014), there was no significant positive correlation between the MIS-D awareness subscale and the MAAS (*r* = 0.108, *p* = 0.117; hypothesis 5a). In addition, the positive correlation between the MIS-D non-judgmental subscale and the MAAS was unexpected, as no attitudinal component of acceptance is covered by the MAAS (*r* = 0.207, *p* = 0.002).

#### Flow Disposition

The positive correlation between the MIS-D refocusing subscale and the FSS (*r* = 0.458, *p* = 0.000; hypothesis 5b) corresponds to the results of the original paper (*r* = 0.43, *p* < 0.001). Contrary to our hypotheses, no further significant positive correlations could be shown in the MIS-D awareness, respectively, non-judgmental subscale and the FSS (*r* = 0.110, *p* = 0.099; *r* = 0.095, *p* = 0.152; hypothesis 5b). Whereas Thienot et al. (2014) found a positive correlation between the awareness subscale and the FSS (*p* < 0.001; *r* = 0.34).

#### Worry and Concentration Disruption

As expected, a negative correlation between the MIS-D non-judgmental subscale and the WAI-T worry subscale could be identified (*r* = −0.265, *p* = 0.011; hypothesis 5c). The same association could be shown between the MIS-D refocusing subscale and the WAI-T concentration disruption subscale (*r* = −0.250, *p* = 0.016; hypothesis 5c). In contrast to Thienot et al. (2014), theoretically expected significant negative correlations between the MIS-D awareness/non-judgmental subscale and the WAI-T concentrations disruption subscale (*r* = 0.076, *p* = 0.472; *r* = −0.139, *p* = 0.187; hypothesis 5c) as well as between the MIS-D awareness/refocusing subscale and the WAI-T worry subscale (*r* = 0.111, *p* = 0.293; *r* = −0.059, *p* = 0.577; hypothesis 5c) could not be shown. In addition, the MIS-D awareness did show an unexpected positive correlation with the WAI-T somatic anxiety subscale (*r* = 0.306, *p* = 0.003).

#### Perfectionism

The theoretically expected negative correlation between the MIS-D non-judgmental subscale and the functional aspect of perfectionism could be supported (*r* = −0.180, *p* = 0.009; hypothesis 5d) as with Thienot et al. (2014), as well as the negative correlation between the MIS-D non-judgmental subscale and the dysfunctional aspect of perfectionism (*r* = −0.229, *p* = 0.001; hypothesis 5d). The expected negative correlation between the MIS-D refocusing subscale and the dysfunctional aspect of perfectionism was only marginally significant (*r* = −0.132, *p* = 0.058; hypothesis 5d).

#### Rumination

Both theoretically expected negative correlations between the two MIS-D subscales non-judgmental, respectively, and refocusing and the RRQ were found (*r* = −0.236, *p* = 0.000; *r* = −0.228, *p* = 0.001; hypothesis 5e).

## Discussion

The purpose of this study was to initially test the reliability (internal consistency: hypothesis 1/test–retest reliability: hypothesis 2), validity (factorial validity: hypothesis 3/convergent validity: hypothesis 5), and measurement invariance across gender and competition type (hypothesis 4a/b) of the MIS-D (German translation) using a total sample of 228 female and male adults out of the authors institutional context.

### Reliability

Adequate reliability estimates of internal consistency (hypothesis 1) were obtained for each subscale of the MIS-D (German version). Although item 7 (awareness) and item 11 (non-judgmental) narrowly missed the critical item-total correlation of 0.30, those items were not excluded (Fisseni, 1997) from further analysis procedures. If we excluded those items, there would have been marginal to no improvement in internal consistency of the MIS-D. In addition, the aim was to preserve the content heterogeneity for substantial convergent constructs. A particularly high reliability and therefore mostly homogeneous tests are often only slightly valid against more complex external criteria. Thus, the primary aim was not to improve the reliability of the test at any cost, but rather to keep the content heterogeneity high (Amelang and Schmidt-Atzert, 2006; Furr, 2021). This proceeding was affirmed by item difficulty indexes of 76.4 (item 7) and 65.3 (item 11), thus, both items were answered in the sense of the characteristic to be measured (Kelava and Moosbrugger, 2011). Results also provide preliminary evidence for test–retest reliability (hypothesis 2) of the MIS-D, which extends the reliability analyses of the original paper by Thienot et al. (2014).

### Factorial Validity

Concerning factorial validity, no one-to-one comparison to the original paper is possible as Thienot et al. (2014) defined a different model in their validation process. In our confirmatory context, the given 19-item MIS-D global model fit was evaluated with particular attention to the absolute fit index RMSEA following Rigdon’s (1996) recommendations to provide a basis for further theory development. In addition, the construction of confidence intervals around particular point estimates allows for a higher informative content and can also prevent freeing additional parameters in a model for the sake of marginal improvements in fit (Browne and Cudeck, 1992). As a consequence, the three-factor model structure was confirmed in the CFA with good model fit (RMSEA ≤0.05, SRMR ≤0.08; hypothesis 3). Therefore, conspicuous regression weights (<0.40) in item 7 and item 11 were no sufficient indication for the removal of these items as well as taking possible suppression effects into account. This potential explanation, which we did not pursue further, indicates that both items can increase the predictive contribution of one or even more other variable(s) by suppressing irrelevant variance components for the prediction (MacKinnon et al., 2000). Due to the salient meaning of the RMSEA, CFI and TLI values, that are marginal below a sufficiently acceptable fit, played only a subordinate role in the evaluation and adjustment of the model, as well as the fact, that the CFI has proven to be appropriate in more exploratory contexts (Rigdon, 1996).

The detailed inner-method convergent analysis showed deviating values in the average variance extracted (AVE) and the indicator reliability (IR) exceeding cutoff values of 0.50 and 0.40, respectively. AVE values below 0.50 do not convey sufficient variance for the items to converge into a single construct, which means that items do not adequately measure the latent construct. There seems to be more error variance than explained variance. Nevertheless, Fornell and Larcker (1981) state, that an AVE less than 0.50 can still be adequate with a composite reliability higher than 0.60. Reliability estimators based on structural equation modeling are typically referred to as composite reliability like the estimated McDonald’s Omega. In contrast to Cronbach’s alpha, this approach claims to include larger estimates of true reliability as construct loadings or weights are allowed to vary (Peterson and Kim, 2013). Composite reliability values (McDonald’s Omega) of all three subscales are higher than 0.60 and therefore confirm adequate AVE values in our study (Fornell and Larcker, 1981). Generally, the indicator reliability (IR) reflects the degree of explanation of the indicator variance by a construct. Even if the IR is not sufficient (<0.40), more indicators improve reliability and validity as there is more information to estimate the latent construct’s shared variance. In order to keep any additional information to the model and in view of a good overall quality measured by composite reliability, there is no cogent reason to exclude any indicators.

Considering options of further modifications of the MIS-D, especially to meet the demand of briefer versions in order to decrease response burden and increase the number of constructs to be measured in parallel, we would like to point out the option of genetic algorithms like Noetel et al. (2019a) applied to the original MIS (Thienot et al., 2014). This approach represents an innovative method to abbreviate questionnaires, but as large modifications could effect the theoretical integrity of a model resulting in a new instrument, an independent sample is required and should therefore be taken under consideration for future research.

### Measurement Invariance Across Gender and Competition Type

Based on the locally confirmed three-factor model, nested model comparisons provide weak evidence for the measurement invariance of the MIS-D (German translation) across competition type (hypothesis 4b) and strong evidence across gender (hypothesis 4a). Consequently, on a construct level, it can be assumed that there is no difference between female and male, which could not be shown in the original paper. Nevertheless, results should be treated with caution since sample size is partially unbalanced between groups, as well as sample size is rather small (<100) in two subgroups (male: *n* = 63/competition: *n* = 92). This might lead to over-rejection of correct models in some measures of absolute model fit, like the RMSEA. Fit indices less-sensitive to sample size like the alternative fit indices (AFIs) should be considered for further instrument refinements. Nevertheless, partial invariance or non-invariance can be also quite informative as given in the grouping variable competition type (Putnick and Bornstein, 2016). Competitive athletes usually have a certain sport-specific expertise and get in touch with psychological training during their career. Thus, some athletes might have already developed skills like goal setting, positive self-talk, or imagery which in consequence may result in a different understanding and interpretation of the items (Grossman, 2008). In order to get to the bottom of this result, exploring mean differences and intercorrelations of the individual items can be helpful to inform development of a refined measure in future research. To evaluate measurement invariance in future studies and to maintain that dynamic and informative aspect of the functioning of a construct across groups, we propose an enlarged and representative sample with the option of the additional grouping variable *sports type* (individual vs. team sport athletes; see Thienot et al., 2014) as well as taking invariance across measurement occasions into account. In doing so, it would also be possible to construct an additional state measure of the MIS-D that could be used in evaluating momentary effects of mindfulness trainings in sports. Furthermore, and according to latent state–trait theory (Steyer et al., 1999, 2015), the consistency and specificity of the proposed State and the existing Trait MIS scales could be determined and thus the extent to which trait and state variance are measured with the respective instruments (see, e.g., Renner et al., 2018; Hock et al. submitted for current empirical demonstrations regarding anxiety and depression).

### Convergent Validity

Concerning convergent validity, it should be kept in mind, that possible deviations reported in this and the original Thienot et al. (2014) paper may be ascribed to mostly different German questionnaires compared to those used by Thienot et al. (2014). There is one exception, namely, the tendency to be mindful in daily life, which was assessed by the MAAS in both versions. In addition, not each instrument measuring the reference constructs was explicitly validated using an athlete population, which might explain different distributions.

#### Mindful Trait in Daily Life

A non-judgmental and open attitude toward upcoming experiences is not specifically part of the MAAS; thus, the positive correlation with the MIS-D non-judgmental subscale was theoretically unexpected. Nevertheless, higher MAAS scores are associated with a higher level of mindful attitude which may also lead in a reduced evaluative attitude toward any occurring experience (Michalak et al., 2008). This mediating effect might be a possible explanation for the positive correlation, as it was also found by Thienot et al. (2014). The less prevailing result in the relationship between the MIS-D awareness subscale and the MAAS (hypothesis 5a) might be due to different content domains of awareness. The MAAS puts stronger emphasize on the cognition and perceiving part of the present moment, whereas items of the MIS-D subscale cover awareness mostly with regard to emotions and bodily sensations.

#### Flow Disposition

Mindfulness is often suggested as a catalyst for flow consisting of a feeling of enhanced physical and psychological functioning with an absence of negative thoughts and self-conscious evaluating, as well as the ability to be absorbed in the present moment task (Csikszentmihalyi, 1978; Jackson and Csikszentmihalyi, 1999; Scott-Hamilton et al., 2016). Anchored in the requirement profile of endurance athletes, a mindful skill like refocusing and acceptance may be especially relevant for this target group as they tend to experience prolonged exposure to sensations of discomfort (Scott-Hamilton et al., 2016). As only a quarter of our total sample (*N* = 228) can be clearly attributed to endurance sports, this might have caused ambiguous results in the MIS-D awareness and non-judgmental subscale (hypothesis 5b). Instead, the strong expected correlation between the MIS-D refocusing subscale and the FSS (hypothesis 5b) illustrates the importance of an attention regulation component in the MIS-D as described by Bishop et al. (2004) in the beginning.

#### Worry and Concentration Disruption

The unexpected positive correlation between the MIS-D awareness subscale and the WAI-T somatic anxiety subscale might be explained by the strong physical reference in their items. Whereas the somatic anxiety subscale describes the physically noticeable component of anxiety, which manifests itself in signs of anxiety, such as palpitations, clammy hands, or a sinking feeling in the stomach (Brand et al., 2009), the MIS-D awareness subscale is defined as “the ability to closely observe one’s internal experience like cognitions, emotions or bodily sensations in the present moment” (Thienot et al., 2014, 73–74). Additionally, mindful awareness implies a higher level of processing, a so-called meta-awareness, to reach a state of distant observation from the self, which may help the athletes to perceive their current state and internal events without responding with sustained evaluation and to detach from non-targeted sensations (Wells, 2005). In consequence, this correlation might be caused by semantic confusion while assessing mindfulness processes as Thienot et al. (2014) already elaborated. Therefore, some items rely on the reader’s experience in mindfulness training to understand the item in its deeper meaning (Grossman, 2008). This phenomenon can also be attributed to the unexpected correlations between the MIS-D awareness and the WAI-T worry/concentration disruption subscales. Nevertheless, the importance of an attention regulation component is clearly shown by the correlations between the MIS-D non-judgmental subscale and the WAI-T worry subscale (hypothesis 5c) and between the MIS-D refocusing subscale and the WAI-T concentration disruption subscale, respectively, (hypothesis 5c). This pattern was also confirmed by Thienot et al. (2014). Contrary to the results in this paper, Thienot et al. (2014) found correlations between the MIS-D non-judgmental subscale and the SAS-2 concentration disruption subscale and between the MIS-D refocusing and SAS-2 worry subscale. At this point, however, it should be mentioned that the concentration disruption subscale showed in both papers (SAS-2/WAI-T) skewness and kurtosis outside of the normality values. Therefore, the interpretation of the given results should be taken with caution.

#### Perfectionism

Within the sports setting, there is still a lack of research integrating the relationship between mindfulness and perfectionism. Hill et al. (2018) showed in their meta-analytical review of multidimensional perfectionism evidence for a maladaptive effect for athletes in the dysfunctional aspect, whereas the functional aspect seems to be more complex and ambiguous. A recent study by van Dyke et al. (2021) claims for a person-centered approach in the context of mindfulness and perfectionism as high-performance quality came along with varying mindfulness and perfectionism techniques. The current findings in the sporting setting underline the complexity of integrating both constructs. The aforementioned ambiguous effects of the functional perfectionism on athletes also seem to be reflected in the results within mindfulness skills in athletes among all subscales of the MIS-D. Nevertheless, the expected negative correlation between the MIS-D non-judgmental subscale and the F-MPS functional component (hypothesis 5e) could be confirmed, even though the basis for this assumption lies outside the sporting context (Hinterman et al., 2012). The theoretically expected negative correlations between the MIS-D subscales and the dysfunctional component of perfectionism could only be confirmed in the non-judgmental subscale (hypothesis 5d). The absence of self-critical and evaluative thinking representing the non-judgmental subscale might explain the negative correlation with the dysfunctional component characterized by concerns over mistake, fear of negative social evaluation, or negative reaction to imperfection (Hill et al., 2018). However, this interpretation must be taken cautiously in both perfectionism components as they also display skewness outside the normality values considered as acceptable.

#### Rumination

The convergent validity results in this reference construct align with theoretically expected negative correlations (hypothesis 5f), as well as with the results of the original paper by Thienot et al. (2014), highlighting again the importance of the attention regulation component and the ability to inhibit elaborative processes in the context of mindfulness in sports (Bishop et al., 2004).

## Conclusion

Mindfulness plays an increasingly important role in sport, whether as a mediator, moderator, or predictor of sport performance as well as the fact, that mindfulness-based training programs are meanwhile highly established in the sports performance setting. Consequently, a reliable and valid instrument to pursue the variety of research questions, for example to test the effectiveness of these interventions, is needed for different languages. The results of this study show that the German version of the Mindfulness Inventory for Sport (MIS-D) is sufficiently reliable. Factorial validity and invariance of measurement across gender and competition type could be largely shown. In addition, the MIS-D subscales correlate with five conceptually related constructs and thus initial evidence for convergent validity is provided. However, current results should be interpreted with caution in terms of preliminary evidence since corresponding data were assessed using partially rather small subsamples. Thus, replications should primarily focus on a larger sample size. In addition, for measurement invariance analyses further relevant subgroups as well as taking invariance across measurement occasions (e.g., invariance across time) should be taken into consideration as a construct can also change over time. On an applied level, the MIS-D can contribute to evaluate mindfulness-based interventions as well as accompany and document the development process of mindfulness-based skills in athletes. By assessing the athletes’ use of mindfulness as a self-regulatory skill when facing disruptive stimuli, the MIS-D offers the possibility to get a better insight into mindfulness-based interventions in the sports context than other common instruments devised for use in clinical settings.

## Data Availability Statement

The raw data supporting the conclusions of this article will be made available by the authors, without undue reservation.

## Ethics Statement

The studies involving human participants were reviewed and approved by Ethikkommission UniBw (Institutional Review Board of the Universität der Bundeswehr). The patients/participants provided their written informed consent to participate in this study.

## Author Contributions

All authors listed have made a substantial, direct, and intellectual contribution to the work and approved it for publication.

## Conflict of Interest

The authors declare that the research was conducted in the absence of any commercial or financial relationships that could be construed as a potential conflict of interest.

## Publisher’s Note

All claims expressed in this article are solely those of the authors and do not necessarily represent those of their affiliated organizations, or those of the publisher, the editors and the reviewers. Any product that may be evaluated in this article, or claim that may be made by its manufacturer, is not guaranteed or endorsed by the publisher.

## Footnotes

^1^1 = *gar nicht*; 6 = *sehr*.

^2^Die nachfolgenden Aussagen beschreiben einige Gedanken und Gefühle, die Sportlerinnen und Sportler kurz vor oder während einer sportlichen Leistungssituation erleben können. Bitte klicken Sie bei jeder Aussage diejenige Antwortmöglichkeit an, die am besten beschreibt, welche Gedanken und Gefühle Sie selbst kurz vor oder während einer sportlichen Leistungssituation im Allgemeinen erleben.

^3^1 = *fast immer*; 6 = *fast nie*.

^4^1 = *trifft nicht zu*; 7 = *trifft zu*.

^5^1 = *gar nicht*; 4 = *sehr*.

^6^1 = *trifft überhaupt nicht zu*; 6 = *trifft sehr gut zu*.

^7^1 = *stimme überhaupt nicht zu*; 5 = *stimme voll und ganz zu*.
