# Supplementary material for: Psychometric Properties of the Mindfulness Inventory for Sport (German Version)
Source: Front Psychol. 2022 May 24;13:864208. doi: 10.3389/fpsyg.2022.864208 (PMC9174664; doi:10.3389/fpsyg.2022.864208)
Supplement: Supplementary file 1 [file Presentation_1.pdf]

Appendix A

Items of the German Version of the Mindfulness Inventory for Sport

Awareness subscale

1. Ich kann die Intensität der Nervosität in meinem Körper wahrnehmen.
2. Ich bin aufmerksam gegenüber dem Typ von Emotionen, die ich empfinde.
3. Ich nehme die Gedanken, die mir durch den Kopf gehen, bewusst wahr.
4. Ich achte auf meine Emotionen, wenn etwas Unerwartetes passiert.
5. Ich kann Gefühle der Aufregung in meinem Körper wahrnehmen.
6. Ich bemerke, wenn ich mir darüber Sorgen mache, was passieren könnte.
7. Ich kann körperliche Beschwerden ausfindig machen, wenn ich diese bemerke/erlebe.
8. Ich kann Ablenkung direkt wahrnehmen.

Non-judgmental subscale (reverse-scored)

9. Wenn mir bewusst wird, dass ich über das Gesamtresultat nachdenke, mache ich mir Vorwürfe, dass ich mich nicht auf relevante Leistungsaspekte fokussiere.
10. Wenn mir bewusst, dass ich sehr aufgebracht bin, da ich gerade verliere, rüge ich mich selbst für die Reaktion.
11. Wenn mir bewusst wird, dass ich sehr aufgeregt bin, da ich gerade gewinne, denke ich, dass es schlecht ist, dieses Gefühl von Aufregung zu haben.
12. Wenn mir bewusst wird, dass ich mich nicht auf meine eigene Leistung konzentriere/fokussiere, rüge ich mich selbst für die Ablenkung.
13. Wenn mir bewusst wird, dass ich über eine vergangene Leistung nachdenke, kritisiere ich mich selbst dafür, dass ich mich nicht auf meine momentane Leistung fokussiere/konzentriere.
14. Wenn ich merke, dass ich mich wegen einem Fehler über mich selbst ärgere, kritisiere/tadele ich mich selbst für diese Reaktion.

Refocusing subscale

15. Wenn mir bewusst wird, dass ich angespannt bin, bin ich schnell in der Lage meine Aufmerksamkeit wieder auf das Eigentliche zurückzulenken.
16. Wenn ich bemerke, dass ich mir Gedanken darüber mache, wie müde ich bin, kann ich meiner Aufmerksamkeit entgegenlenken und mich wieder auf das Eigentliche konzentrieren.
17. Wenn mir bewusst wird, dass ich mich nicht auf meine eigene Leistung fokussiere, bin ich schnell in der Lage meine Aufmerksamkeit wieder auf die Dinge zurückzulenken, die mir zur einer guten Leistung verhelfen.
18. Wenn ich bemerke, dass einige meiner Muskeln schmerzen, kann ich mich schnell wieder neu auf das Eigentliche konzentrieren.
19. Wenn mir bewusst wird, dass ich sehr aufgeregt bin, weil ich gerade gewinne, bleibe ich trotzdem auf das Eigentliche fokussiert.

Appendix B

Inter-item correlation matrix

**Items**	**01**	**02**	**03**	**04**	**05**	**06**	**07**	**08**	**09**	**10**	**11**	**12**	**13**	**14**	**15**	**16**	**17**	**18**	**19**
**01**	1.00																		
**02**	0.39	1.00																	
**03**	0.13	0.42	1.00																
**04**	0.20	0.43	0.36	1.00															
**05**	0.54	0.33	0.26	0.28	1.00														
**06**	0.26	0.31	0.19	0.14	0.42	1.00													
**07**	0.02	0.16	0.11	0.24	0.16	0.11	1.00												
**08**	0.11	0.20	0.30	0.27	0.17	0.18	.031	1.00											
**09**	-0.08	-0.09	-0.11	-0.18	-0.11	-0.20	-0.08	-0.22	1.00										
**10**	-0.11	-0.03	0.05	-0.09	-0.10	-0.09	0.10	-0.02	0.33	1.00									
**11**	-0.19	0.06	0.13	-0.13	-0.08	0.05	-0.10	0.01	0.06	0.25	1.00								
**12**	-0.14	-0.04	0.01	-0.10	-0.14	-0.10	-0.06	-0.05	0.27	0.37	0.33	1.00							
**13**	-0.04	-0.05	-0.09	-0.01	-0.01	-0.11	-0.04	-0.12	0.41	0.33	0.15	0.51	1.00						
**14**	-0.10	-0.14	-0.08	-0.11	-0.04	-0.15	-0.02	-0.04	0.31	0.28	0.13	0.25	.052	1.00					
**15**	0.02	0.05	0.08	0.02	0.02	-0.03	0.17	0.01	0.14	-0.03	0.03	-0.01	0.00	-0.03	1.00				
**16**	0.10	0.16	0.05	0.02	0.12	0.17	0.14	0.08	-0.07	-0.04	-0.05	0.03	-0.05	-0.14	0.32	1.00			
**17**	-0.03	0.07	0.11	0.10	0.05	0.00	0.13	0.05	0.04	-0.02	-0.03	0.01	-0.01	0.03	0.61	0.43	1.00		
**18**	-0.01	0.06	0.13	0.19	0.17	0.00	0.24	0.12	-0.14	-0.06	0.03	-0.05	-0.04	-0.08	0.38	0.25	0.40	1.00	
**19**	0.11	0.09	0.06	0.01	0.21	0.06	0.04	0.08	0.05	-0.04	0.12	-0.03	-0.01	0.04	0.43	0.24	0.38	0.27	1.00

Note. Item 01-08 = Awareness subscale. Item 09-14 = Non-judgmental subscale. Item 15-19 = Refocusing subscale.
